# Evaluation of expression and function of vascular endothelial growth factor receptor 2, platelet derived growth factor receptors-alpha and -beta, KIT, and RET in canine apocrine gland anal sac adenocarcinoma and thyroid carcinoma

**DOI:** 10.1186/1746-6148-8-67

**Published:** 2012-05-25

**Authors:** Bridget K Urie, Duncan S Russell, William C Kisseberth, Cheryl A London

**Affiliations:** 1 The Ohio State University College of Veterinary Medicine, Department of Veterinary Clinical Sciences, 601 Vernon L Tharp Street, Columbus, OH, 43210, USA; 2 The Ohio State University College of Veterinary Medicine, Department of Veterinary Biosciences, 1900 Coffey Road, Columbus, OH, 43210, USA

**Keywords:** Canine, Anal sac adenocarcinoma, Apocrine gland of the anal sac, Thyroid carcinoma, Toceranib

## Abstract

**Background:**

Toceranib phosphate (Palladia) has a reported objective response rate of 25% in both canine apocrine gland anal sac adenocarcinoma (AGASACA) and thyroid carcinoma (TC), with stable disease occurring in an additional 50-60% of dogs. The basis for the observed responses to toceranib is not known. The purpose of this study was to evaluate AGASACA and TC samples for the expression and activation of VEGFR2, PDGFRα, PDGFRβ, KIT and RET to assess whether dysregulation of these receptor tyrosine kinases (RTKs) may contribute to the biologic activity of toceranib.

**Results:**

mRNA for VEGFR2, PDGFRα/β, KIT and RET was detected in all AGASACA samples. mRNA for VEGFR2, PDGFRα/β, and KIT was detected in all TC samples, while mRNA for RET was amplified in 10/15 samples. No phosphorylation of VEGFR2, PDGFRα/β, or KIT was observed on the arrays. However, phosphorylation of RET was detected in 54% of the primary AGASACA and 20% of TC. VEGFR2 was expressed in 19/24 primary and 6/10 metastatic AGASACA and 6/15 TC samples. KIT was present in 8/24 primary and 3/10 metastatic AGASACA and 9/15 TC samples. PDGFRα expression was noted in all tumor samples. In contrast PDGFRβ expression was found in only a few tumor samples but was evident in the stroma of all tumor specimens.

**Conclusions:**

Known targets of toceranib are expressed in both AGASAC and TC. Given the observed expression of VEGFR and PDGFRα/β and phosphorylation of RET, these RTKs merit investigation as to their roles in the biology of AGSACA and TC and their contribution to toceranib’s activity.

## Background

Toceranib phosphate (Palladia®; Pfizer Animal Health, Madison, NJ, USA) is an oral oxindole multi-targeted receptor tyrosine kinase (RTK) inhibitor (TKI) that blocks the activity of VEGFR2, PDGFRα,/β, FMS-like tyrosine kinase 3 (FLT-3), stem cell factor receptor (KIT), and colony stimulating factor receptor (CSFR1) [1]. It was approved for the treatment of canine mast cell tumors (MCTs) based on a single agent response rate of approximately 43% in dogs with recurrent or non-resectable grade 2 or 3 MCTs [2]. Toceranib also exhibited activity against multiple tumor types in the original phase 1 study, suggesting that the action of toceranib against receptors other than KIT may play a role in the responses observed in solid tumors [3]. In support of this, toceranib’s kinome mirrors that of sunitinib (Sutent®, Pfizer Inc, New York, NY), a very closely related multi-targeted TKI that has demonstrated activity against renal cell carcinoma, gastrointestinal stromal tumors, thyroid carcinomas, and pancreatic neuroendocrine tumors in humans [4-6]. The biologic activity of sunitinib in these solid tumor settings has been attributed to its inhibition of VEGFR2, RET (another RTK) and likely PDGFRα/β [4,5,7].

A retrospective analysis of the use of toceranib in canine solid tumors provided further support for possible biologic activity against multiple tumor types including apocrine gland anal sac adenocarcinoma (AGASACA), metastatic osteosarcoma, thyroid carcinoma (TC), carcinomas of the head and neck, and nasal carcinoma [8]. In that study, of the 32 dogs with AGASACA that were treated with toceranib, a partial response (PR) to therapy as defined by RECIST criteria was noted in 8/32 (25%) dogs and 20/32 dogs had stable disease (SD, 62.5%) for a clinical benefit (CB) rate of 87.5%. Of the 15 dogs with thyroid carcinoma, PR was observed in 4/15 (26.7%) dogs and 8/15 (53.5%) experienced SD for a CB rate of 80%. These data support the notion that targets of toceranib may be expressed and functional in these tumor types.

In humans, thyroid neoplasia has been associated with point mutations in BRAF and RAS and rearrangements in RET and PAX8/peroxisome proliferators-activated receptor γ (PPAR γ) [9-12]. Greater than 70% of papillary thyroid carcinomas harbor mutations in BRAF, RAS, or RET/PTC resulting in activation of the mitogen-activated protein kinase pathway [13] and roughly 75% of follicular thyroid carcinomas have mutations in either RAS or PAX/PPARγ [10]. Recognition of these mutations led to the use of targeted therapeutics in the multimodal treatment of thyroid tumors in people. Clinical trials evaluating multi-targeted TKIs in the setting of progressive and/or metastatic disease have exhibited PRs in 14-49% of patients, with 35 to 73% of patients exhibiting SD for times exceeding 6 months [11,14]. The multi-targeted TKIs sunitinib, sorafenib, motesanib and pazopanib all inhibit VEGFR2, PDGFRα/β, and KIT and share similar response rates, yet it remains to be elucidated inhibition of which target is responsible for the observed biologic activity. Given the broad range of target inhibition exhibited by these drugs, it is possible that the clinical benefits are due to effects exerted on multiple receptors rather than on one particular target.

In contrast to the relatively well-described molecular profiles of human tumors, the potential targets for therapeutic intervention in canine cancers have not been well-characterized.

This is particularly relevant for both AGASACA and TC;, as there has been only one study that investigated the expression of PDGFRβ and KIT in AGASACA [15]. The expression of other RTKs targeted by toceranib was not examined. Another study evaluated dogs with familial medullary thyroid carcinoma for RET dysregulation similar to that found in human familial tumors but failed to identify any mutations in the gene after complete sequencing [16]. Given that toceranib has biologic activity against both AGASACA and TC, the purpose of this study was to evaluate these tumors for the expression of VEGFR2, PDGFRα/β, KIT and RET at both the message and protein level to begin to dissect the molecular basis for its observed activity.

## Results

### Sample demographics

Tumor samples from primary and metastatic AGASACA were collected from a heterogenous population of dogs that were presented to the Ohio State University Veterinary Medical Center (OSU-VMC). The mean age was 9.9 years (range 7.4 to 13.9, median of 10.6 years). Eleven of the dogs were spayed females, and 13 were castrated males. Mixed breed dogs were overrepresented (n = 11) with the remaining 13 dogs being defined as a discernable breed and included Labrador retriever (n = 4), and one dog representing each of the following breeds: Shetland sheepdog, Siberian husky, German shepherd dog, boxer, Lhasa apso, miniature schnauzer, cocker spaniel, golden retriever and English setter. Ten of the 24 dogs were hypercalcemic at the time of diagnosis. Nine dogs staged free of gross metastatic disease while 15 dogs had evidence of regional lymph metastasis at the time of diagnosis. Of the 15 dogs with lymph node metastasis, one dog had bilateral AGASACA and one dog also had evidence of pulmonary metastatic disease.

TC tumor samples were collected from 15 dogs that were presented to the OSU-VMC. The mean age was 9.5 years (range 6 to 14, median of 10.0 years). Ten of the dogs were castrated males, four were spayed females, and one was an intact male. The most commonly represented breeds included Labrador retriever (n = 6) and mixed breed (n = 4), with one dog representing the following breeds: Golden retriever, pit bull terrier, bichon frise, blue tick hound, and a Norfolk terrier. Five of the tumors were hypersecretory, and all were diagnosed as follicular thyroid carcinoma based on histopathology. Two dogs had pulmonary metastasis and one dog had a metastatic lymph node at the time of diagnosis. All 15 tumors were deemed amenable to surgical removal.

### Phosphoprotein arrays

**Figure 1:**
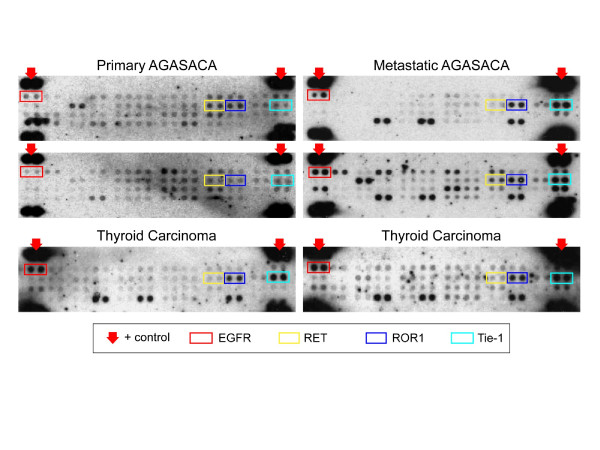
**Phospho-RTK array profiling of canine AGASACA and TC tumors.** Shown are representative examples of phosphoprotein arrays of paired primary and metastatic AGASACA and TC using the Proteome Profiler Human Phospho-RTK Array Kit. This platform allowed simultaneous screening of 42 different RTKs. Determination of phosphorylation was based on comparison of capture antibody of interest to positive controls located on the periphery of the array. On these sample arrays, positive controls, EGFR, RET, ROR-1, and Tie-1 have been identified for comparison

The Proteome Profiler™ Human Phospho-RTK Array Kit provided a platform to assess phosphorylation of 42 different receptor tyrosine kinases in primary and metastatic AGASACA tissue specimens and primary thyroid carcinoma samples using the available flash frozen tumor specimens. Representative examples of the RTK arrays are shown in Figure 1 and a summary of the array results for the AGASACA and TC samples is provided in Table 1. Greater than 50% of the primary AGASACAs demonstrated phosphorylation of EGFR (67%), Dtk/TYRO3 (87%), Ron (54%), RET (54%) ROR1 (92%), ROR2 (62%), while phosphorylation of FGFR3 (38%) insulin-R (50%), Tie-1 (21%) and Tie-2 (25%) was observed in a smaller percentage of samples.

**Table 1:** Phosphoprotein screening results from AGASACA and TC tumor samples

**RTK**	**Primary AGASACA n (%)**	**Metastatic AGASACA n (%)**	**Thyroid carcinoma n (%)**
EGFR	16 (67)	7 (64)	14 (100)*
Dtk/TYRO3	21 (87)	3 (27)	8 (53)
Insulin-R	12 (50)	1 (9)	6 (40)
ROR-1	22 (92)	11 (100)	15 (100)
ROR-2	15 (62)	-	-
Ret	13 (54)	1 (9)	3 (20)
Tie-1	5 (21)	11 (100)	15 (100)
Tie-2	6 (25)	1 (9)	6 (40)
Ron	13 (54)	1 (9)	5 (30)
FGFR3	9 (38)	1 (9)	-

All of the metastatic lymph node specimens showed phosphorylation of ROR1 and Tie-1, with phosphorylation of EGFR (64%) also observed in a majority of the samples. Table 2 summarizes differences in RTK phosphorylation between paired primary and metastatic AGASACA samples. The TC samples demonstrated phosphorylation of primarily EGFR (100%), Dtk/TYRO3 (53%), ROR1 (100%) and Tie-1 (100%), with insulin-R (40%), and RET (20%), Tie-2 (40%) and Ron (30%) occurring less frequently.

**Table 2:** Phosphoprotein screening results from paired AGASACA samples

**RTK**	**Primary AGASACA n (%)**	**Metastatic AGASACA n (%)**
EGFR	5 (45)	7 (64)
Dtk/TYRO3	10 (91)	3 (27)
Insulin-R	4 (36)	1 (9)
ROR-1	11 (100)	11 (100)
Ret	5 (45)	1 (9)
Tie-1	1 (9)	11 (100)
Tie-2	1 (9)	1 (9)
Ron	7 (64)	1 (9)
FGFR3	4 (63)	1 (9)

### Reverse transcriptase -polyermase chain reaction

While the phosphorylation array represents a useful screening tool to assess tissue samples for activation of key RTKs, it does not detect total protein, and will therefore not give a complete picture as to the extent of tumor RTK expression. To provide an initial assessment of expression of the RTKs, total RNA was extracted from normal anal sac and thyroid glands, primary and metastatic AGASACA and primary TC tissue samples, and RT-PCR was performed for canine VEGFR2, KIT, RET, PDGFRα and PDGFRβ. Message for all of these RTKs was detected in all AGASACA tissue samples and paired metastatic lymph nodes. Interestingly, message for RET was not detected in the normal anal sac tissue. Similar to AGASACA, message for VEGFR2, Kit, PDGFR-α and PDGFR-β was detected in all TC samples, while message for RET was detected in only 10/15 tumors. Normal thyroid tissue samples expressed message for all of the RTKs.

### Immunohistochemistry

**Figure 2:**
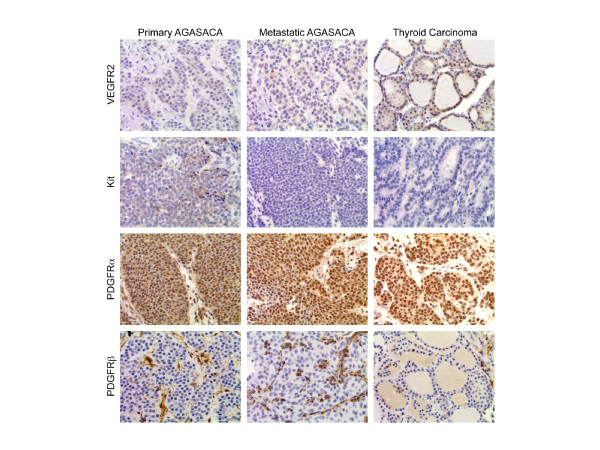
**Immunohistochemistry for VEGFR2, KIT, and PDGFR α/β.** The TMA constructed for primary and metastatic AGASACA and TC were probed for VEGFR2, KIT, PDGFRα, and PDGFRβ. Positive immunoreactivity was scored and location of staining was noted. Shown are representative images for primary and metastatic AGASACA and TC for each RTK evaluated

Core samples from all tumor tissues except one metastatic AGASACA lymph node were available for evaluation, and tissue microarrays were constructed to evaluate expression of receptors of interest in all samples (Tables 3,4,5,6 and Figure 2). Positive immunoreactivity for VEGFR2 was noted in the cytoplasm of tumor cells in 19/24 primary and 6/10 metastatic AGASACA and 6/15 TC samples. Positive immunoreactivity for KIT in the cytoplasm of tumor cells was noted in 8/24 primary and 3/10 metastatic AGASACA and 9/15 TC samples. Strong cytoplasmic and stromal staining for PDGFRα was noted in all primary and metastatic AGASACA and all of the TC samples. Cytoplasmic PDGFRβ expression was noted in 4/24 primary and 1/10 metastatic AGASACA and 4/15 TC tumor cells, while intense stromal staining was noted in all tumor samples. The normal anal sac samples exhibited positive immunoreactivity for VEGFR2 and PDGFRα while normal thyroid samples displayed positive reactivity for VEGFR2, PDGFRα and KIT. Unfortunately, RET expression could not be evaluated on this platform due to the lack of a validated antibody for use in canine formalin-fixed paraffin embedded samples.

**Table 3:** RTK expression in primary AGASACA samples by IHC

**RTK**	**Negative**	**+**	**++**	**+++**	**Predominant localization**
VEGFR2	4 (17)	6 (25)	2 (8)	11 (46)	C
PDGFRα	0	0	0	24 (100)	N, C
PDGFRβ	20 (83)	3 (13)	1 (4)	0	M
Kit	16 (67)	3 (13)	2 (8)	3 (13)	C

C: cytoplasmic; M: membranous; N: nuclear.

**Table 4:** RTK expression in metastatic AGASACA samples by IHC

**RTK**	**Negative**	**+**	**++**	**+++**	**Predominant localization**
VEGFR2	4 (40)	1 (10)	0	5 (50)	C
PDGFRα	0	0	0	10 (100)	N, C
PDGFRβ	9 (90)	1 (10)	0	0	M
Kit	7 (70)	0	2 (20)	1 (10)	C

C: cytoplasmic; M: membranous; N: nuclear.

**Table 5:** RTK expression in TC samples by IHC

**RTK**	**Negative**	**+**	**++**	**+++**	**Predominant localization**
VEGFR2	13 (87)	1 (7)	0	1 (7)	C
PDGFRα	0	0	0	15 (100)	C
PDGFRβ	11 (73)	1 (7)	2 (13)	1 (7)	C
Kit	6 (40)	1 (7)	4 (270	4 (27)	C

C: cytoplasmic; M: membranous; N: nuclear.

**Table 6:** Stromal RTK expression in AGASAC and TC samples by IHC

**RTK**	**Primary AGASACA n (%)**	**Metastatic AGASACA n (%)**	**Thyroid carcinoma n (%)**
VEGFR2	0	1 (10)	0
PDGFRα	24 (100)	10 (100)	15 (100)
PDGFRβ	24 (100)	10 (100)	15 (100)
Kit	0	0	0

## Discussion

The purpose of this study was to evaluate AGASACA and TC for the expression of VEGFR2, PDGFRα/β, Kit and Ret at both the message and protein level to begin to dissect the molecular basis for the observed response of these tumors to toceranib. While the messages for VEGFR2, Kit, PDGFRα and PDGFRβ were detected in all tumor samples, only PDGFR-α and VEGFR2 expression were detected in all AGASACA and TC tumor cells by IHC. Strong expression of PDGFRβ was observed in the stroma of both tumor sets. While message for RET was identified in all tumor samples, this RTK was found to be phosphorylated in only 54% of AGASACA and 20% of TC samples. Similarly, VEGFR2, PDGFRα and PDGFRβ were detected in all tumor specimens by IHC; however,phosphorylation of these was not observed on the arrays. These data suggest that while these RTKs may be present in most AGASACAs and TCs, most probably they do not exist in a state of continual activation/signaling observed in the setting of typical RTK dysregulation associated with mutation, chromosomal translocation, and over-expression. As such, the majority of SD/CB in dogs with AGASACA and TC may relate to inhibition of RTKs that are in support of tumor growth (i.e., VEGFR2 and PDGFRβ associated with vascular endothelium and stroma) rather than a direct inhibition of specific RTKs expressed on tumor cells.

To date, no driver mutations or chromosomal alterations have been reported in either canine AGASACA or TC. A report of a canine pedigree with familial medullary TC (MTC) that clinically paralleled MTC in humans failed to demonstrate a mutation in RET [16]. Another study identified the presence of somatic mutations in p53 in canine TC [17]. Aside from the well documented relationship between AGASACA and parathyroid hormone related- protein, limited data exists regarding the genetics and molecular biology of this tumor [18-20]. With regard to AGASACA, a higher frequency of the disease has been documented in English Cocker Spaniels with the DLA-DQB1 allele [21], and a direct relationship between E-cadherin expression assessed by IHC on formalin fixed samples and survival in dogs with AGASACA has been documented [22]. As previously mentioned, a previous study of 77 archival AGASACA samples found 19.5% expressed positive immunoreactivity for PDGFRβ and 2.6% expressed positive immunoreactivity for KIT [15]. Clearly, more detailed investigations of the molecular aberrations in both AGASACA and TC are needed to better characterize the key biological drivers of these diseases.

In contrast to the lack of published data in veterinary medicine, significant strides have been made in understanding the molecular biology of differentiated thyroid cancer (DTC) in people, with specific gene signatures and tumor-initiating events having been identified [23]. DTC includes papillary TC (PTC), follicular TC (FTC), and Hurthle cell carcinoma (HCC). Although often grouped together, there are molecular differences among PTC, FTC and HCC that are associated with phenotype and biological behavior. For example, alterations in several RTKs have been identified, with the best described being dysregulation of RET present in 5-30% of sporadic PTC in adults [24,25]. Chromosomal rearrangements and mutations resulting in ligand-independent constitutive activation of RET induce neoplastic transformation of thyroid follicular cells through the activation of multiple intracellular signal transduction pathways [26]. Dysregulation of NTRK1, BRAF, Ras, and PAX/PPARγ has also been associated with the development and progression of other thyroid cancers [24,27].

In the present study, message for RET was detected in the majority of samples and evidence of phosphorylated protein was detected in 20% of TC and 54% of primary AGASACA. In the paired primary and metastatic AGASACA samples, 5 of the primary tumors had evidence of phosphorylated RET as detected by the phospho-RTK arrays, while it was only detected in one of the lymph nodes suggesting down-regulation of the protein or loss of phosphorylation following metastasis. Unfortunately, an antibody for probing of canine RET for IHC has yet to be validated and limited the authors’ ability to assess protein expression on the TMA.

Activating mutations in RET have been documented in human PTC and MTC, multiple endocrine neoplasia 2 syndromes, pheochromocytoma, and paragangliomas that are known to drive tumor cell growth [28][29]. There is also evidence accumulating to support RET activation and signaling in the pathogenesis of a subset of estrogen receptor α positive breast cancer [30] and overexpression of RET has been reported in prostate, pancreatic, neural crest derived tumors, bile duct carcinomas, melanoma, and lung cancers [31-37]. VEGFR and RET exhibit structural similarity and as such, TKIs that target VEGFR such as sunitinib, motesanib and vandetanib have been demonstrated to also inhibit RET phosphorylation [5,27]. Sunitinib, a TKI with an inhibitory profile very similar to that of toceranib, has been used as therapy for progressive/metastatic DTC in people resulting in modest objective responses but promising CB with prolonged periods of disease stabilization [5,14,27] A recent kinome analysis of toceranib demonstrated that RET is a target of this TKI (London et al, unpublished) suggesting that as is the case with several human carcinomas, phosphorylation of RET in AGASACA and TC from some dogs may be driving cell growth and survival and could therefore be responsible for the objective response to therapy noted in a subset of dogs on toceranib.

The role of PDGFR in thyroid cancer pathogenesis has also been investigated [38]. In one study, 8 patients with advanced DTC exhibiting overexpression of PDGF receptors based on IHC were treated with imatinib, a TKI that targets Bcr-Abl, PDGFR and KIT [38]. Partial responses were documented in 2/8 with another 4/8 experiencing SD for a CB of 75% suggesting that PDGFR may represent a target for therapeutic intervention. In the current study, we found PDGFRα to be expressed in all tumor cells of all canine TC samples, while PDGFRβ was primarily expressed in the tumor stroma by IHC. Interestingly, phosphorylation of these receptors was not documented using the phospho-RTK array, indicating that while expressed, PDGFRα/β is likely not driving tumor growth and survival through constitutive activation.

Similar to the case of TC, we identified expression of PGDFRα in the tumor cells of all AGASACA samples and PDGFRβ in the tumor stroma by IHC. Expression of PDGFRβ in tumor cells was restricted to only 16% of tumor samples, which is consistent with a previous study that found 15 of 77 samples to be positive for this receptor [15]. The association of PDGFRβ primarily with the stroma, and not tumor cells, in both AGASACA and TC suggests that toceranib may be exerting much of its biologic activity through effects on the tumor stroma and blood supply, particularly in cases where SD is observed.

In our study, message for KIT was detected in all primary and metastatic AGASACA, as well as TC tumor samples. In contrast, KIT protein immunoreactivity as assessed by IHC was found in only 8/24 primary and 3/10 LN AGASACA samples and 9/15 of the TC samples, with expression being localized to tumor cells, not stroma. The discordant results between message and protein expression may be secondary to detection of KIT message present in mast cells and other inflammatory cells present within the tumor. Our results also differ from a previous study in which only 2/77 AGASACA samples were positive for KIT by IHC. The reason for this discordance is unclear, although differences in tissue processing, primary antibody used and staining protocol may be contributory. Nevertheless, phosphorylation of KIT was not detected on the phospho-RTK arrays suggesting that as with PDGFRβ, KIT signaling is probably not playing a role in the driving tumor cell growth and proliferation of AGASACA and TC in dogs. This is consistent with the biology of thyroid neoplasia in people in which KIT dysregulation is not believed to be a contributory factor in tumor biology.

Message for VEGFR2 was detected in tumor cells from all AGASACA and TC samples evaluated. While most (19/25 primary and 6/10 LN metastases) expressed VEGFR2 protein as assessed by IHC, only 2/15 TC samples were positive for protein. As with PDGFR and KIT, there was no evidence of VEGFR2 phosphorylation on the phospho-RTK arrays. While stromal VEGFR2 expression was only noted in one AGASACA LN sample, this may be due to the fact that VEGFR2 is primarily expressed on circulating endothelial precursors and neo-vessels, rather than mature blood vessels, and as such it may be difficult to identify these populations in the formalin-fixed specimens. However, the role of VEGFR2 and angiogenesis in TC is supported by several observations including the high degree of vascularity of thyroid tumors, the correlation between increased VEGF expression and development of metastatic disease, and evidence revealing that VEGFR2 inhibition may contribute biologic responses observed with multi-targeted RTK inhibitor therapy in humans [39].

The phospho-RTK arrays identified evidence of phosphorylation of several other RTKs that may be influential in the development and progression of both AGASACA and TC. Tie-1 and Tie-2 comprise the Tie family of RTKs and share the angiopoietins as activating ligands. Tie-1 is typically expressed on vascular endothelium and has been implicated in tumor angiogenesis [40]. Expression of Tie-1 has been reported in breast, gastric, colon, and thyroid cancers, but constitutive activation has only been proven in a breast cancer cell line [40,41]. In a study of 135 patients with TC, Tie-1 immunoreactivity was observed in PTC and anaplastic TC but not FTC or follicular adenomas, and it was also noted that large tumors had decreased expression [41]. Based on the recognized biological differences between PTC and FTC, the difference in Tie-1 expression may influence the lymph node metastases that are commonly observed in PTC [41]. Tie-2 expression has been shown to be increased with breast cancer and is also expressed in human PTC, FTC and follicular adenomas [42,43]. A correlation has been demonstrated between Tie-2 expression and increased risk of metastasis and relapse in breast cancer patients, and expression is associated with decreased survival [42]. Although the role of Tie-2 in thyroid cancer has not been clearly delineated, it is thought to be associated with cellular proliferation [43]. While the roles of both Tie-1 and Tie-2 in AGASACA and TC are currently unknown, further investigation is warranted given the observed phosphorylation of both receptors in many tumor samples in this study.

It is well recognized that EGFR dysregulation is a powerful oncogenic driver with overexpression and activating mutations documented in numerous tumors of epithelial origin [44]. While expression of EGFR has been evaluated in canine mammary tumors, brain tumors, nasal carcinomas, and lung tumors, it has yet to be proven as a driver of tumor proliferation in any canine cancer [45]. A recent report evaluating several markers in human thyroid carcinoma found strong EGFR expression in invasive PTC and FTC [46]. Vandetanib, a TKI that inhibits EGFR as well as VEGFR and RET, is approved for the treatment of advanced MTC but the impact of EGFR inhibition in this disease is unknown and to date, there is limited data about its activity in the treatment of DTC, although clinical trials are presently underway [5,27]. EGFR expression was not evaluated in the present study, but evidence of phosphorylation supports further investigation.

## Conclusions

The descriptive nature of this project has inherent limitations. Protein expression by IHC does not directly correlate with a causative role in tumor growth and survival, and while phosphorylation of several key RTKs was observed on the arrays, their exact contribution to AGASAC and TC was not investigated. The tumor samples in this study were not microdissected, and thus included both tumor and stroma in protein lysates and RNA as well as non-neoplastic cell infiltrates such as mast cells or residual lymph node. Nevertheless, the findings of VEGFR and PDGFR protein expression and phosphorylation of RET merit further investigation as to their roles in the biology of AGSACA and TC as well as to their contribution to the observed response to toceranib.

## Methods

### Tissue samples

Tumor tissue samples were collected from clinical cases that presented to the OSU-VMC. Consent for tissue collection was obtained from all owners in accordance with an approved IACUC protocol (2010A0015) and collected by the OSU-VMC Biospecimen Repository. A total of 24 primary AGASACA with 11 paired metastatic regional lymph nodes and 15 primary TC were identified. Surgical and post-mortem collected tumor samples were snap frozen in liquid nitrogen and stored at −80°C. Tissue samples were also placed in formalin and processed for routine paraffin embedding for histopathology. The medical records of all dogs were reviewed and data pertaining to signalment, staging, treatment, and survival were abstracted.

### Protein lysate preparation and phosphoprotein arrays

To assess relative phosphorylation of 42 different RTKs in primary tumor samples, the Proteome Profiler™ Human Phospho-RTK Array Kit was used (R&D Systems, Minneapolis, MN, USA). Briefly, frozen tumor samples were pulverized using a frozen mortar and pestle. The resulting powder was resuspended in liquid nitrogen, and transferred to 1.5 mL microcentrifuge tube. Once the liquid nitrogen evaporated away, the samples were allowed to thaw on ice and resuspended in tissue lysis buffer containing 20 mM Tris–HCl, 2 mM EDTA, 137 mM NaCl, 10 μg/mL aprotinin, 10 μg/mL leupeptin, 1 mM sodium orthovanadate, 1% IGEPAL® CA 630, and 10% glycerol. Samples were rocked for 1 hour at 4°C, centrifuged for 15 minutes at 14,000 RPM at 4°C, and supernatants collected. Bradford protein quantification assay was performed on the extracts using BioRad Reagent (BioRad, Hercules, CA, USA). 100 μg of protein lysate was used to perform the RTK array following the manufacturer’s instructions.

### RNA isolation and RT-PCR

Powdered sample of homogenized tissue (described above) was placed in 500uL of TRIzol (Invitrogen Corporation, Carlsbad, CA) and stored at −80°C until RNA isolation according to the manufacturer’s protocol. RNA was quanitated with the Nanodrop-100 spectrophotometer (Thermo Scientific, Wilmington, DE, USA). SuperScriptIII (Invitrogen Corporation, Carlsbad, CA) reverse transcriptase was used for reverse transcription as previously described [47]. For the PCR reactions, primers directed against canine genes of interest (Table 7) were designed to span an intron and were used to amplify product from the cDNA. The program for each PCR reaction was unique for each primer set and was designed to generate a single PCR product. Taq polyermase (Promega, Madison, WI) was used for each reaction. The amplified PCR products were separated by agarose gel electrophoresis, stained with ethidium bromide and visualized with ultraviolet light. All PCR products were verified by sequencing at The OSU Shared Resources Nucleic Acid Core Laboratory.

**Table 7:** RT-PCR primer sets for canine RTKs

**RTK**	**Primers**	**Product size (bp)**
c-Kit	232 F: 5’ -- GAG AAC ACA CAC AAC GAA TG – 3’ 414R: 5’ -- GCA GCG GAC CAG CGT ATC ATT G – 3’	185
PDGFRα	1917 F: 5’ – GCT CTC ATG TCG GAA CTG AAG – 3’ 2152R: 5’ – GTG TGC TGT CAT CAG CAG G – 3’	237
PDGFRβ	2235 F: 5’ – GAC GAG TCA GTG GAT TAC GTG – 3’ 2562R: 5’ – GTC TCT CAT GAT GTC ACG AGC CAG – 3’	329
c-Ret	206 F: 5’ – GCC ACT GTG ATG CTG TAG AGA GCA G – 3’ 259R: 5’ – GTG CGG CAG AGC TCA TCA CAC AGT GG – 3’	210
VEGFR2	1537 F: 5’ – GTA AGT ACC CTT GTT ATC CAA GCA GCC – 3’ 1728R: 5’ – CGT AGT TCT GTC TGC AGT GCA CCA C – 3’	195
GAPDH	526 F: 5’ – GTC CAT GCC ATC ACT GCC ACC CAG – 3’ 718R: 5’ – CTG ATA CAT TGG GGG TGG GGA CAC – 3’	196

### Tissue microarray construction and immunohistochemistry

Representative areas of tumor tissue were identified on hematoxylin-eosin (HE) stained sections by a single pathologist (DR) for triplicate core sampling. Cores were extracted from the corresponding areas of paraffin embedded blocks and inserted into predetermined sites on the TMA recipient block. Immunohistochemical staining was performed for VEGFR2 (Santa Cruz Biotechnology; 1:10 dilution) [48], PDGFRα (Santa Cruz Biotechnology; 1:50) [49], PDGFRβ (Biogenex Labs; 1:200) [15], and KIT (Dako; 1:400), on all ASACA and TC samples. Negative controls consisted of irrelevant isotype matched antibody at matched dilutions. Both the construction of the TMA bloc and immunohistochemical staining were performed by the OSU- CVM Veterinary Biosciences Histopathology Laboratory.

### Scoring of immunoreactivity

Subjective quantitative scoring of positive or negative immunoreactivity of cells and stroma within the cores were performed by three of the investigators (WK, DR, BU). Percentage of neoplastic cells staining positive was scored as follows: <5% = 0, 5-25% = 1, 26-50% = 2, and >50% =3. Location of staining was also noted as cytoplasmic (C), membranous (M), nuclear (N), and stromal (S).

## Abbreviations

VEGFR2: Vascular endothelial growth factor receptor-2/KDR/Flk-2; PDGFRα: Platelet derived growth factor receptor-alpha; PDGFRβ: Platelet derived growth factor receptor-beta; KIT: Stem cell factor receptor; RET: Rearranged during transfection; receptor for glial cell line-derived neurotrophic factor; RTK: Receptor tyrosine kinase; AGASACA: Apocrine gland of the anal sac adenocarcinoma; TC: Thyroid carcinoma; RT-PCR: Reverse-transcriptase polymerase chain reaction; TMA: Tissue microarray; IHC: Immunohistochemistry.

## Competing interests

The authors declare no financial or non-financial competing interests.

## Authors’ contributions

BU conceived the study and performed sample preparation, phosphoprotein arrays, RT-PCR, TMA template, and IHC scoring. DR evaluated all histopathology samples and participated in TMA construction and IHC scoring. WK participated in IHC scoring and contributed to project coordination. CL’s contributions were essential for project design and coordination as well as manuscript preparation. All authors read and approved the final manuscript.
